# Characterization of a STAT-1 Knockout Mouse Model for Machupo Virus Infection and Pathogenesis

**DOI:** 10.3390/v17070996

**Published:** 2025-07-16

**Authors:** Stephanie R. Monticelli, Ana I. Kuehne, Russell R. Bakken, Susan R. Coyne, Kenise D. Lewis, Jo Lynne W. Raymond, Xiankun Zeng, Joshua B. Richardson, Zebulon Lapoint, Jennifer L. Williams, Christopher P. Stefan, Jeffrey R. Kugelman, Jeffrey W. Koehler, Andrew S. Herbert

**Affiliations:** 1 Viral Immunology Branch, United States Army Medical Research Institute of Infectious Diseases, Frederick, MD 21702, USA; stephanie.r.monticelli.ctr@health.mil; 2 The Geneva Foundation, Tacoma, WA 20817, USA; ana.i.kuehne.civ@health.mil (A.I.K.); russell.r.bakken.civ@health.mil (R.R.B.); 3 Developmental Diagnostics Branch, United States Army Medical Research Institute of Infectious Diseases, Frederick, MD 21702, USA; susan.r.coyne.civ@health.mil (S.R.C.); christopher.p.stefan.civ@health.mil (C.P.S.); 4 Chenega Corporation, Anchorage, AK 99503, USA; kenise.d.lewis.ctr@health.mil; 5 Pathology Division, United States Army Medical Research Institute of Infectious Diseases, Frederick, MD 21702, USA; jolynne.w.raymond.civ@health.mil (J.L.W.R.); xiankun.zeng.civ@health.mil (X.Z.); 6 Cherokee Nation Integrated Health, Catoosa, OK 74015, USA; joshua.b.richardson2.civ@health.mil (J.B.R.); zebulon.r.lapoint.ctr@health.mil (Z.L.); jennifer.l.williams410.ctr@health.mil (J.L.W.); 7 Center for Genome Science, United States Army Medical Research Institute of Infectious Diseases, Frederick, MD 21702, USA; jeffrey.r.kugelman.mil@health.mil; 8 Operational Diagnostics Branch, United States Army Medical Research Institute of Infectious Diseases, Frederick, MD 21702, USA; jeffrey.w.koehler4.civ@health.mil

**Keywords:** arenavirus, STAT1, Machupo virus, arenavirus mouse models

## Abstract

Machupo virus (MACV), a member of the *Arenaviridae* family and causative agent of Bolivian hemorrhagic fever, results in lethality rates of 25–35% in humans. Mice lacking the signal transducer and activator of transcription 1 (STAT-1^−/−^) have previously been shown to succumb to MACV infection within 7–8 days via the intraperitoneal route. Despite these reports, we observed partial lethality in STAT-1^−/−^ mice following challenge with wild-type MACV. Serial sampling studies to evaluate the temporal progression of infection and pathologic changes after challenge revealed a two-phase disease course. The first phase was characterized by viral load and pathological lesions in the spleen, liver, and kidney followed by a second, lethal phase, defined by high viral titers and inflammation in the brain and spinal cord resulting in neurological manifestations and subsequent mortality. Tissue adaptation in the brains of challenged STAT-1^−/−^ mice resulted in a fully lethal model in STAT-1^−/−^ mice (mouse-adapted; maMACV). A similar two-phase disease course was observed following maMACV challenge, but more rapid dissemination of the virus to the brain and overall pathology in this region was observed. The outcome of these studies is a lethal small rodent model of MACV that recapitulates many aspects of human disease.

## 1. Introduction

Machupo virus (MACV), the etiological agent of Bolivian hemorrhagic fever (BHF), is a member of the *Arenaviridae* family [1,2,3]. First described during an outbreak of severe febrile disease in the Beni district of Bolivia in 1959, recurring outbreaks have been reported sporadically, and the number of human cases has been increasing since 2006 [4,5,6]. Infection is primarily associated with rodent urine or excreta spread through the *Calmys callosus* vector, or large vesper mouse, and generally occurs through inhalation of aerosolized virus, ingestion of virus through contaminated food, introduction onto mucous membranes, and on occasion through nosocomial transmission [1,2,3,7,8,9,10,11].

The incubation period for BHF varies from 5 to 21 days, but averages 7 to 14 days following exposure [2,5,11,12,13,14]. Infection in humans often follows a biphasic disease progression [5]. An initial prodromal phase is characterized by fever, lethargy, malaise, dehydration, headache, myalgia, dehydration, anorexia, and cough, which is followed by a neurologic and hemorrhagic phase that leads to hemorrhage of mucosal membranes, petechiae, hypotension, blood in vomit and stool, tremors, convulsions, muscle spasms, encephalopathy, melena, and, in severe cases, death [5,14]. The mortality rate of BHF is estimated to be around 30% [5,15]. Those that recover may experience a convalescence period of several weeks to months signified by fatigue, dizziness, hair loss, and general weakness. There are currently no approved therapeutics or vaccines available to treat or prevent infection by MACV. Use of convalescent immune sera and plasma has shown some efficacy against MACV in human infection if administered prior to the establishment of hemorrhagic disease [6,11]; however, no published clinical trials exist to confirm the efficacy of this treatment. Similarly, immune serum has been shown to be protective in animal studies [16,17]. Ribavirin, a wide-spectrum antiviral, has also been tested in a limited number of patients; however, no clinical trials have been performed to test efficacy in humans [18,19].

Several animal models of MACV disease have been described. Inoculation of non-human primates (NHPs), including African green monkeys, rhesus monkeys, and cynomolgus macaques, results in a similar biphasic disease course as observed in humans [20,21,22,23,24]. An initial acute phase manifests approximately 7–10 days post exposure, with death occurring approximately 8–25 days post virus exposure. This acute phase is followed by a second phase where surviving NHPs exhibit severe neurological manifestations that can result in mortality. Further, the Chicava strain of MACV has been shown to be lethal in Hartley guinea pigs without adaptation by serial passage [21,25]. Detailed analysis of the disease course and pathogenesis in this model show many similar clinical signs and patterns of infection and subsequent inflammation in both humans and NHPs [25]. This includes initial nonspecific symptoms that progress to neurological conditions manifesting on day 16–20 and subsequent lethality. Adult immunocompetent mice are resistant to MACV infection [21]. However, mice with a compromised interferon (IFN) pathway have been shown to be susceptible to infection. Infection of IFN-αβ/γ receptor double knockout (KO) mice via the intraperitoneal (IP) route results in a biphasic disease course characterized by significant weight loss 10–14 days post-challenge followed by neurological impairment and mortality 20–40 days post-viral challenge [26]. MACV-challenged IFN-αβ/γ KO mice maintain high viral loads in the brains that persist through death. Mice lacking signal transducer and activator of transcription 1 (STAT-1^−/−^) have also been reported to be susceptible to MACV infection, succumbing approximately 6–20 days post-challenge depending on the route of challenge [27]. In this model, infection of mice via the IP route resulted in a single phase of disease with complete lethality by 7–8 days post-challenge and a lack of any neurological manifestations.

Here, we aimed to validate the STAT-1^−/−^ model of MACV infection with the primary goal of utilizing it to support the evaluation of MACV-specific medical countermeasures. However, our preliminary studies revealed a contradictory disease course in comparison to that observed in prior reports and notably looked similar to the biphasic disease presentation observed in other MACV rodent and NHP models. As such, the objective of the present study was to further characterize the disease course and pathogenesis of MACV disease in STAT-1^−/−^ mice.

## 2. Materials and Methods

### 2.1. Cell Lines

VeroE6 (RRID: CVCL-0574) were obtained from the American Type Culture Collection (ATCC). Cells were cultured in Dulbecco’s Modified Eagle Medium (DMEM) (ThermoFisher Scientific, Waltham, MA, USA) with glutamine, 10% heat-inactivated fetal bovine serum (ΔFBS; Gibco), and 1% penicillin–streptomycin (ThermoFisher Scientific, Waltham, MA, USA). All in vitro infections were conducted using DMEM with glutamine, 2% ΔFBS, and 1% penicillin–streptomycin. Cells were maintained in a 37 °C incubator supplied with 5% CO_2_. Cell lines were not authenticated following purchase.

### 2.2. Animal Models

The animals used were 4–12-week-old male and female B6.129S(Cg)-*Stat1*^tm1Dlv^/J mice (STAT-1^−/−^; strain #012606; The Jackson Laboratory, Bar Harbor, ME, USA), ranging in weight from 15 to 30 g, in all animal challenge experiments. These animals had previously never undergone experimentation and were confirmed to be free of contaminating bacterial or viral pathogens by the vendor. Animals were randomly allocated to experimental groups and provided with food and water ad libitum and housed in individually ventilated cages in groups of 5–10 mice per cage. Murine challenge studies were conducted under an Institutional Animal Care and Use Committee-approved protocol in compliance with the Animal Welfare Act, Public Health Service Policy, and other Federal statutes and regulations relating to animals and experiments involving animals. The facility where this research was conducted (USAMRIID) is accredited by AAALAC International and adheres to principles stated in the Guide for the Care and Use of Laboratory Animals, National Research Council 2011. Mice determined to be moribund, in accordance with the USAMRIID IACUC approved criteria, were promptly euthanized. Mice were housed under specific pathogen-free conditions at USAMRIID.

### 2.3. In Vivo Challenges

The 4–12-week-old male and female STAT-1^−/−^ mice (The Jackson Laboratory, Bar Harbor, ME, USA) were exposed IP or intranasally (IN), where indicated, to 100, 1000, or 5000 pfu (where indicated in the results and figure legends) of MACV (strain as indicated). The mice were observed daily for clinical signs of disease and morbidity and weighed. The mice were scored on a 4-point grading scale, where a 1 was defined by decreased grooming and/or ruffled fur, a 2 defined by subdued behavior when un-stimulated, a 3 defined by lethargy, hunched posture, and/or subdued behavior even when stimulated, and a 4 defined by bleeding, unresponsiveness, severe weakness, or inability to walk. Mice scoring a 4 were considered moribund and were euthanized. Neurological manifestations including circling, head tilt, and partial or full hind limb paralysis did occur and were considered in the scoring criteria when they impacted overall behavior as described above. Mice with partial or full hind limb paralysis that could not access food or water were considered moribund.

### 2.4. Serial Sampling Studies

For serial sampling studies, at the indicated days post-challenge (0, 2, 4, 6, 8, 10, 12, 14, 16, 18, 20, 24, 28, 36, 41, 45, and 63 days or 0, 2, 4, 6, 8, 10, 12, 14, 16, 17, and 18 days post wild-type MACV-Chicava or maMACV-Chicava challenge, respectively), the mice (*n* = 2–5) were euthanized and whole blood, liver, spleen, kidney, lung, testis, brain, and spinal cord were collected. The mice were sampled randomly.

### 2.5. Generation of Mouse-Adapted MACV

For each passage, STAT-1^−/−^ mice were exposed IP to 1000 pfu of challenge stock, and a subset (*n* = 2–5) was harvested for spleen or brain tissue at various times post-challenge. The spleen and brain were harvested at time points corresponding to the first and second phase of disease, respectively. The spleen was harvested 6–8 days post-challenge, and the brain was harvested approximately 18–30 days post-challenge at times when the mice were displaying severe weight loss and clinical signs of disease (clinical score ≥ 3). The remaining mice (*n* = 5–8) were monitored for lethality for 42 days post-challenge. Tissues were homogenized and viral titers were assessed by a plaque assay. Spleen and brain homogenate from one to two mice with the highest viral titers were combined separately to generate spleen and brain homogenate challenge stocksrespectivelyT for use in the next passage. In total, four passages were undertaken. Four stocks were determined to be more lethal than parental isolates; MACV-Carvallo or MACV-Chicava passaged one time in the brains of STAT1^−/−^ mice (P1 brain homogenate), or two times in the spleens of STAT1^−/−^ mice (P2 spleen homogenate). Cell culture-derived stocks of in vivo passaged virus were generated by the infection of VeroE6 cell monolayers with mouse-adapted tissue homogenate at a multiplicity of infection (MOI) of 0.01. Monolayers were incubated at 37 °C for 1 h. Following infection, inoculum was removed, fresh media were added, and cells were incubated for 5 days at 37 °C. Following incubation, cell culture supernatant was collected and clarified at 4000× *g* for 10 min. Clarified supernatant was collected, and a viral titer was assessed by plaque assay.

### 2.6. Plaque Assay

Whole blood was centrifuged at 12,000× *g* for 10 min to collect serum. Tissues were homogenized in DMEM supplemented with glutamine, 2% ΔFBS, and 1% penicillin–streptomycin in gentleMACS^™^ M tubes (Miltenyi Biotec, Gaithersburg, MD, USA) to generate a 10% tissue homogenate and clarified to remove cellular debris by centrifugation at 4000× *g* for 10 min. Serial dilutions of serum and tissue homogenates were prepared in DMEM supplemented with glutamine, 2% ΔFBS, and 1% penicillin–streptomycin. Following dilutions, 200 µL from each dilution was inoculated onto VeroE6 cell monolayers in 6-well plates. After adsorption for 1 h at 37 °C, cell monolayers were overlaid with a mixture of 1 part 1% agarose (Seakem ME, Rockland, ME, USA) and 1 part 2× Eagle basal medium, 30 mM HEPES buffer, and 2% ΔFBS. Plates were incubated for 5 days at 37 °C, and after 5 days a second overlay supplemented with 5% neutral red was added. Plaques formed by infectious MACV were then counted 24 h later. Titers are shown as plaque-forming units (PFUs)/mL.

### 2.7. ELISA

High-binding half-area plates (Greiner Bio-One, Monroe, NC, USA) were coated with 5 µg/mL of recombinant MACV GP (Mapp Biopharmaceuticals, San Diego, CA, USA) and incubated overnight at 4 °C. Approximately eighteen hours later, plates were blocked with blocking buffer (5% milk in 1× Phosphate-buffered saline [PBS] with 0.05% Tween-20; PBST) for two hours at an ambient temperature. The serum was diluted 1:10, 1:100, 1:300, and 1:1000 in blocking buffer; following blocking, liquid was removed by flicking, and each serum dilution was added to plates in duplicate and incubated for two hours at an ambient temperature. Plates were washed 3× with PBST and then incubated with either horseradish peroxidase (HRP)-conjugated goat anti-mouse IgG (Jackson ImmunoResearch Laboratories, West Grove, PA, USA) or HRP-conjugated goat anti-mouse IgM (Jackson ImmunoResearch Laboratories, West Grove, PA, USA) for one hour at an ambient temperature. Plates were washed 3× with PBST and incubated with ABTS [2,2′-azino-bis(3-ethylbenzothiazoline-6-sulfonic acid)] substrate for 15 min at ambient temperatures. Plates were read at 405 nm (SpectroMax M5; Molecular Devices, San Jose, CA, USA).

### 2.8. Neutralization Assay

Serum samples were heat inactivated at 56 °C for 30 min. MACV-Chicava and MACV-Carvallo were incubated with serial 3-fold dilutions of heat-inactivated serum (starting at a dilution of 1:5) for 1 h at 37 °C. The serum–virus mixture was added to monolayers of VeroE6 cells in a 96-well plate at a final multiplicity of infection of 0.6 (MACV-Chicava) or 0.5 (MACV-Carvallo) and incubated for 1 h at 37 °C. The infection medium was removed, and a fresh cell culture medium without serum was added. Then, 48 h post-infection, the culture medium was removed, and plates were fixed in 10% neutral buffered formalin (Valtech, Pottstown, PA, USA) for at least 24 h at 4 °C. The plates were removed from formalin and permeabilized with 0.2% Triton-X for 10 min at room temperature and treated with blocking buffer (5% milk). Infected cells were detected by sequential incubation with anti-mouse MACV-specific GP antibody (Creative Diagnostics, Shirley, NY, USA; CABT-B8948) and secondary detection antibody goat anti-mouse conjugated to AlexaFluor 488 (1:2000 dilution; Invitrogen, Waltham, MA, USA). Percent infection was determined using the Cytation5 high-content imaging instrument, and data analysis was performed using the Gen 5.11 software (BioTek), Winooski, VT, USA.

### 2.9. Cytokine and Chemokine Analysis

Serum cytokine and chemokine levels were measured using the MILLIPLEX^®^ Premixed 32 Plex Mouse Cytokine/Chemokine Magnetic Bead Panel (Sigma-Aldrich, St. Louis, MO, USA) according to the manufacturers protocol. Samples were analyzed on a Luminex MAGPIX^®^ System (ThermoFisher Scientific, Waltham, MA, USA) using xPONENT 4.2 software.

### 2.10. PCR

Tissue samples were homogenized and clarified prior to extraction as required. Samples were inactivated using a 3:1 ratio of TRIzol LS Reagent to sample (ThermoFisher, Waltham, MA, USA). A total of 400 µL of Trizoled material was purified using the EZ1 Virus Mini Kit v 2.0 (Qiagen, Germantown, MD, USA) using the EZ1 Advanced XL robot (Qiagen) according to the manufacturer’s recommendations. The total elution volume was 150 µL. RT-qPCR was performed using TaqPath™ RT-qPCR Master Mix, CG (ThermoFisher, Waltham, MA, USA) on the QuantStudio DX (ThermoFisher, Waltham, MA, USA), with primers and probes targeting the L protein gene of the Machupo virus (Forward 5′-AGGAARAGTGCAGGACTCATTG-3′, Reverse 5′-ATTTGTGAYGAAGATGGCRGT-3′, Probe 6FAM 5′-TGGACCCCATTTTCA-3′ MGNBQ). Reference material was quantified using the QIAcuity Digital PCR System and the QIAcuity One-Step Viral RT-PCR Kit (Qiagen, Germantown, MD, USA) using the same RT-qPCR assay. Samples were run in technical triplicate, and concentrations were calculated using a standard curve of quantified reference material included on each run. Sample concentrations were reported in copies of target per mL and were based on extracted material. Concentrations that fell outside the linear range of the standard curve were extrapolated. The limit of detection of the assay based on the standard curve is approximately 10 copies/µL.

### 2.11. Sequencing

For sequence confirmation and contamination detection, each strain was subject to next-generation sequencing on the Illumina MiSeq platform (Illumina, Inc., San Diego, CA, USA). First, RNA from samples was extracted using the Purelink RNA mini kit (Invitrogen, Waltham, MA, USA). Isolated RNA was amplified using a sequence-independent, single-primer amplification (SISPA) protocol [28]. Sequencing libraries were prepared using the Illumina DNA Prep kit and sequenced on an Illumina MiSeq using a 600-cycle kit. Sequences were trimmed and quality filtered with Trimmomatic (v.0.39) [29], then mapped to the Machupo virus L (NC_005079.1) and S (NC_005078.1) segments for the Carvallo strains and the Chicava references L (AY624354) or S (AY924202) for the Chicava strains using SeqManNGen (v.17.5.1) and in-house scripts. Sequencing data was also assembled into contigs using ray (v.2.2) and in-house scripts. Each segment for each sample had a coverage greater than 97% (minimum depth: 20 bp) and an average depth of greater than 200×. Up to 3% of the segment sequence with depth less than 20× occurred in the extreme ends of any segment, including the UTR. There were no gaps in the coverage observed internally in any segments. Contigs were serially blasted against the nt database on multiple settings to exclude the presence of contaminating bacteria or viruses from the strains. Sequences were submitted to Genbank, and reference numbers are displayed in Table 1.

### 2.12. Histopathology

Necropsies were performed on each mouse immediately following euthanasia in the USAMRIID biosafety level 4 laboratory. Tissues were fixed by immersion in 10% neutral buffered formalin (Valtech, Pottstown, PA, USA) and held in containment for a minimum of 21 days. Tissues were trimmed, processed, embedded in paraffin, cut by microtomy, stained with hematoxylin and eosin, cover slipped, and screened.

### 2.13. In Situ Hybridization

Tissue sections were placed on positively charged slides and stained by ISH. To detect MACV genomic RNA in formalin-fixed embedded tissues, ISH was performed using the RNAscope 2.5 HD RED kit (Advanced Cell Diagnostics, Newark, CA, USA). Forty ZZ ISH probes targeting MACV genomic RNA of segment L, 541-2716 (GenBank #AY624354.1), were designed and synthesized by Advanced Cell Diagnostics (Cat #11824291-C1). Tissue sections were deparaffinized with xylene, underwent a series of ethanol washes and peroxidase blocking, and were then heated in kit-provided antigen retrieval buffer and digested by kit-provided proteinase. Sections were exposed to ISH target probe pairs and incubated at 40 °C in a hybridization oven for 2 h. After rinsing, the ISH signal was amplified using kit-provided Pre-amplifier and Amplifier conjugated to alkaline phosphatase and incubated with a Fast Red substrate solution for 10 min at room temperature. Sections were then stained with hematoxylin, air-dried, and cover slipped.

### 2.14. Statistical Analysis

For correlation analysis, the R-squared value was calculated by Spearman’s correlation coefficient. Survival outcomes were compared against each group by Mantel–Cox tests and color coded to display which group was compared. Weight loss and clinical outcomes were compared against each group by Analysis of Variance (ANOVA) test. Statistical significance was determined by *p* ≤ 0.05. All statistical analyses were conducted in GraphPad Prism Software V9.5.1.

## 3. Results

### 3.1. MACV Is Partially Lethal in STAT-1^−/−^ Mice

Previous reports indicated that MACV is lethal in STAT-1^−/−^ mice, with mice succumbing to infection 7–8 days post-challenge when the virus was administered by the IP route [27]. To confirm these reports, mice were exposed to 100, 1000, or 5000 plaque-forming units (pfu) of two different isolates of MACV, Chicava, and Carvallo, by the IP route. Initially, mice were sourced from Taconic Biosciences (129S6/SvEv-Stat1^tm1Rds^; model #2045) for consistency with previously published reports that utilized these mice [27]. However, challenge experiments with these mice utilizing both isolates of MACV and at multiple doses resulted in no lethality and a complete absence of any clinical signs of disease in multiple repeat experiments. Slight weight loss was observed, but it was minimal (Supplementary Figure S1).

**Figure 1:**
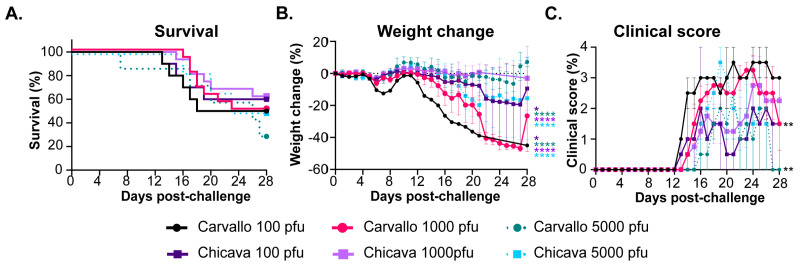
**Wild-type MACV is partially lethal in STAT-1^−/−^ mice.** STAT-1^−/−^ mice (*n* = 7–15) were exposed to the indicated pfu of either MACV-Chicava or MACV-Carvallo via the intraperitoneal route and monitored for (**A**) survival, (**B**) weight loss, and (**C**) clinical score for 28 days post challenge. Statistical significance is represented as * *p* ≤ 0.05, ** *p* ≤ 0.01, **** *p* ≤ 0.0001. Significance is color coded relative to the group to which it is compared.

Subsequent challenge experiments utilized mice from The Jackson Laboratory (B6.129S(Cg)-Stat1^tm1Dlv^/J; strain #12606; hereafter referred to as STAT-1^−/−^). Challenge of these STAT-1^−/−^ mice with either MACV-Chicava or MACV-Carvallo at all doses tested resulted in only partial lethality (defined by either death or euthanasia of moribund mice) of approximately 40–70% with an overall mean time to death [MTD] of 18.06 ± 4.12 (Figure 1A). Minimal differences in lethality were observed between challenge isolates or doses with MTD ranging between 15.6 ± 2.07 and 19.67 ± 3.51. We did not observe a relationship between weight and lethality across the isolates and doses tested (Figure 1B). For example, challenge with 100 or 1000 pfu of MACV-Carvallo resulted in approximately 50% weight loss day 28 post-challenge, but challenge with 5000 pfu of MACV-Carvallo or any dose of MACV-Chicava resulted in only 10–20% weight loss by day 28 post challenge (Figure 1B). Overall, MACV-challenged mice experienced a biphasic course of disease, characterized by an initial transient period of mild disease followed by a prolonged period of severe disease and lethality. The first phase of disease occurred approximately 5–8 days post-challenge and was defined by body weight loss of approximately 10% (Figure 1B) but minimal-to-no observable clinical symptoms of disease (Figure 1C). By day 10 post challenge, mouse weights had returned to baseline but were quickly followed by a second period of substantial weight loss (>10% up to 50%) that persisted through the end of the study (Figure 1B). Throughout the second phase, mice displayed multiple clinical symptoms of disease, including initial symptoms of ruffled fur, hunched posture, and lethargy that were followed by rapidly worsening clinical symptoms that included neurological complications such as head tilt, ataxia, circling, and partial or full hind limb paralysis, with mice succumbing to infection 8 to 28 days post-challenge.

### 3.2. Analysis of the Progression of MACV Disease in STAT-1^−/−^ Mice

#### 3.2.1. Infectious Virus Load

**Figure 2:**
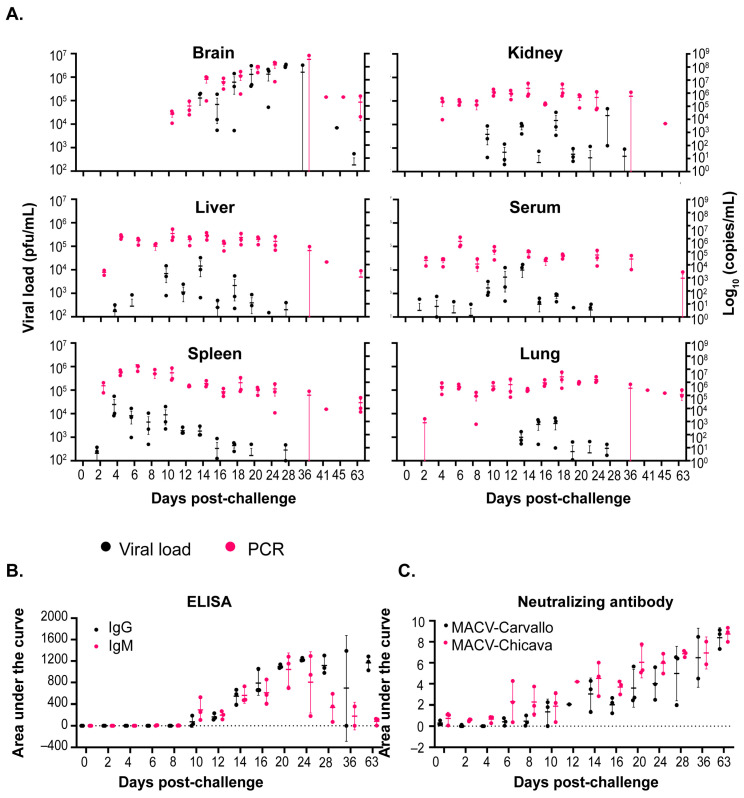
**Disease progression of MACV-Chicava in STAT-1^−/−^ mice.** STAT-1^−/−^ mice were infected with 1000 pfu of MACV-Chicava via the intraperitoneal route, and tissue samples were harvested at the indicated time points following MACV infection. (**A**) Samples were evaluated for infectious virus (viral load; black) and MACV-specific genome copies by PCR (pink). (**B**) IgG (black) and IgM (pink)-specific antibodies and (**C**) neutralizing antibodies against MACV-Carvallo (black and MACV-Chicava (pink) were assessed by ELISA and a microneutralization assay, respectively.

Given the differences in the results reported herein and prior reported disease in STAT-1^−/−^ mice [27], serial sampling studies were performed to investigate the temporal progression and pathogenesis of MACV infection in this model. Due to the availability of guinea pig and non-human primate models of infection for the Chicava isolate compared to the Carvallo isolate [25,30] and similar disease presentation observed between the two isolates in initial experiments (Figure 1), serial sampling studies were completed with only the Chicava isolate. Mice were challenged with 1000 pfu of MACV-Chicava, and on days 2, 4, 6, 8, 10, 12, 14, 16, 18, 20, 24, 28, 36, 41, 45, and 63 post-challenge, 3–5 mice were euthanized and the brain, kidney, liver, lung, spleen, and serum were harvested for viral load analysis by plaque assay and MACV-specific polymerase chain reaction (PCR) (Figure 2A). As expected, a two-phase disease course was observed, with STAT-1^−/−^ mice losing approximately 10–25% of their weight between days 5 and 8 in the first phase, followed by a recovery period and a subsequent second phase characterized by rapid weight loss (Supplementary Figure S2A) and severe clinical signs of disease 15 to 46 days post-challenge (Supplementary Figure S2B). Variability in weight loss and clinical score was observed between individual animals, which was expected due to the model being partially lethal.

Infectious viral titers were first detected in the spleen and serum on day 2 post challenge, followed by detection in the liver on day 4, kidney on day 10, and brain and lung on day 14 post challenge (Figure 2A). While infectious titers in the kidney, liver, lung, spleen, and serum either fell or stayed level (~10^4^ pfu/mL) until the end of this study, infectious titers in the brain rose rapidly at day 14 to 10^5^ pfu/mL and continued to climb, peaking at 10^6^–10^7^ pfu/mL on day 36 post challenge. Infectious titers in the brain were the highest of all the tissues at any time point tested by approximately two logs. Surprisingly, the infectious virus was detected in the brain of one of three mice on day 63 post challenge even though this mouse exhibited minimal weight loss and clinical disease throughout the study (Supplementary Figure S1). Notably, the virus was also detected in the liver, spleen, lung, serum, and kidney by PCR in this animal, in addition to one other mouse 63 days post challenge.

#### 3.2.2. Antibody Titers and Neutralizing Response

An assessment of the IgG- and IgM-specific antibody response was performed by ELISA on serum samples obtained at the above time points (Figure 2B). IgG- and IgM-specific antibodies were first detected on day 10 post challenge. IgM-specific antibodies increased until a peak on day 20 and then fell to almost undetectable levels on day 63 post-challenge. Conversely, IgG-specific antibodies continued to develop until the end of the study on day 63 post challenge. The development of neutralizing antibodies was assessed by microneutralization assays against MACV-Chicava and MACV-Carvallo (Figure 2C). As expected, neutralization potency against the challenge isolate, MACV-Chicava, was enhanced over MACV-Carvallo. Surprisingly, low-level neutralizing titers were observed on day 6 post challenge, prior to the detection of IgM- and IgG-specific antibodies by ELISA and continued to rise through the end of the study. However, differences in assay sensitivity likely account for differences in antibody detection by ELISA and by a neutralization assay [31].

#### 3.2.3. Correlation of Viral Load with Weight Change and Clinical Score

Our serial sampling study showed that infectious virus titers in the liver, spleen, kidney, and lung were detected early after infection, while infectious titers in the brain were detected at later times post challenge, specifically day 14, and persisted through the end of the study. Detection of infectious virus in the brain aligned closely with initiation of the second, lethal phase of disease beginning approximately day 15 post challenge and characterized by significant weight loss (>40%), neurological complications, and subsequent lethality (Figure 1). We hypothesized that dissemination of the virus to the brain would trigger the severe morbidity and subsequent mortality observed in STAT-1^−/−^ mice. To evaluate this further, we calculated the correlation coefficient (R^2^) of infectious viral titer per tissue for each individual mouse plotted against percent weight loss (Table 2) and clinical score (Table 3) at time of euthanasia. We observed a significant positive correlation between infectious brain titer and both weight loss (*p* value < 0.0001; R^2^ = 0.4169; Table 2) and clinical score (*p* value < 0.0001; R^2^ = 0.3427; Table 3). We also observed significant positive correlations between lung titer and weight loss and lung and kidney titers to clinical score; however, the R^2^ values were 1.75-to-3.77-fold less positive than those observed between clinical score/weight loss and brain titers. Taken together, this data shows that the appearance of infectious virus in the brain is positively correlated with the onset of significant disease and morbidity.

#### 3.2.4. Anatomic Pathology

**Figure 3:**
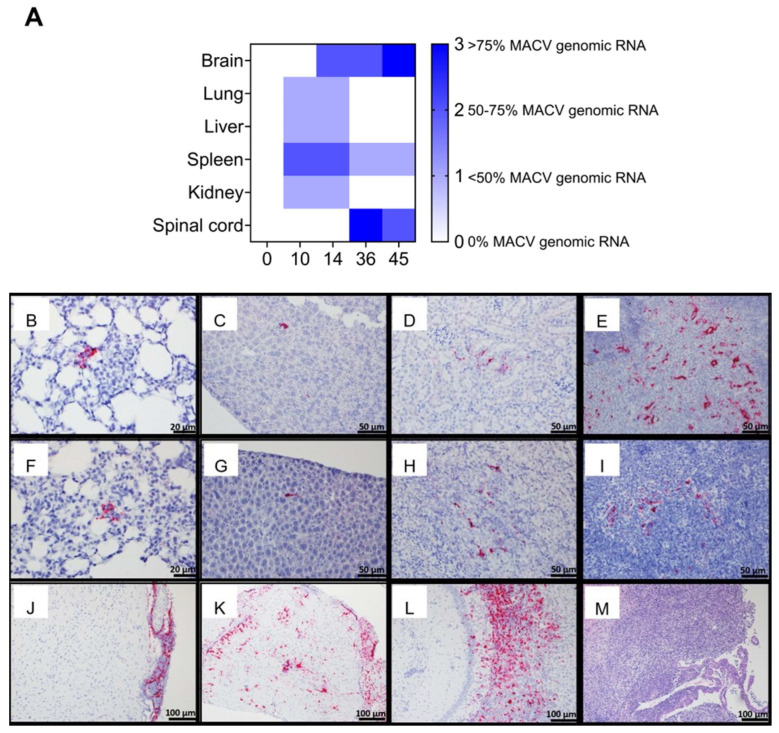
**Pathological changes following MACV challenge in STAT-1^−/−^ mice.** (**A**) Percentage of tissue stained with MACV genomic RNA is plotted. *X*-axis is days post challenge. (**B**–**L**) Representative photomicrographs of in situ hybridization and (**M**) H&E stained sections of (**B**,**F**) lung, (**C**,**G**) liver, (**D**,**H**) kidney, (**E**,**I**) spleen, (**J**,**L**,**M**) brain, and (**K**) spinal cord removed from infected mice at (**B**–**E**) day 10, (**F**–**J**) 14, (**K**,**M**) 36, and (**L**) 45 post challenge.

Tissues were analyzed for the presence of MACV genomic RNA by in situ hybridization (ISH) (Figure 3). MACV-specific RNA was first detected in the lung, liver, spleen, and kidney on day 10 post challenge (Figure 3A–E) and persisted through day 14 (Figure 3F–I). At these time points, detection was minimal in the lung, liver, and kidney, with moderate detection in the red pulp of the spleen. MACV genomic RNA was not detected in the brain until day 14, where it was primarily detected in the meninges (Figure 3J). Days 36 and 45 post exposure, viral antigen was still highly detected in the brain, primarily in the inflammatory areas, and was also observed in the spinal cord, but was undetectable in the liver, lung, and kidney and only minimally detected in the spleen (Figure 3L). Additional hematoxylin and eosin (H&E) staining was conducted on brain tissue collected 45 days post-exposure, which showed necrotizing encephalitis in the brain (Figure 3M). Overall, pathological findings were in accordance with the patterns of infectious virus observed in these tissues (Figure 2).

### 3.3. Adaptation of MACV in STAT-1^−/−^ Mice

With the goal of generating a fully lethal stock of MACV, brain or spleen tissue from STAT-1^−/−^ mice challenged with either MACV-Chicava or MACV-Carvallo were sequentially passaged. To maximize success, tissues were harvested from mice targeting both phases of disease utilizing the serial sampling study to guide selection of tissues and time points for harvest. In each passage, the mice were challenged with 1000 PFU of MACV and spleen or brain were harvested from 2 to 3 mice per time point either 8 days or 20–37 days post-challenge, respectively. Brain tissue was harvested from mice experiencing severe clinical signs of disease (lethargy, hunched posture, weight loss, neurological complications). Tissue was homogenized, clarified of large debris by centrifugation, and viral titers were evaluated by a plaque assay. Tissues from 1 to 2 mice with the highest titer were combined and used to subsequently challenge naïve STAT-1^−/−^ mice for the next passage round. Spleen and brain homogenates were passaged separately. Serial passaging for each MACV isolate was performed four times. To monitor success of the adaptation, STAT-1^−/−^ mice (*n* = 5–7 per round) were challenged and monitored for weight loss, clinical score, and mortality for 40 days.

**Figure 4:**
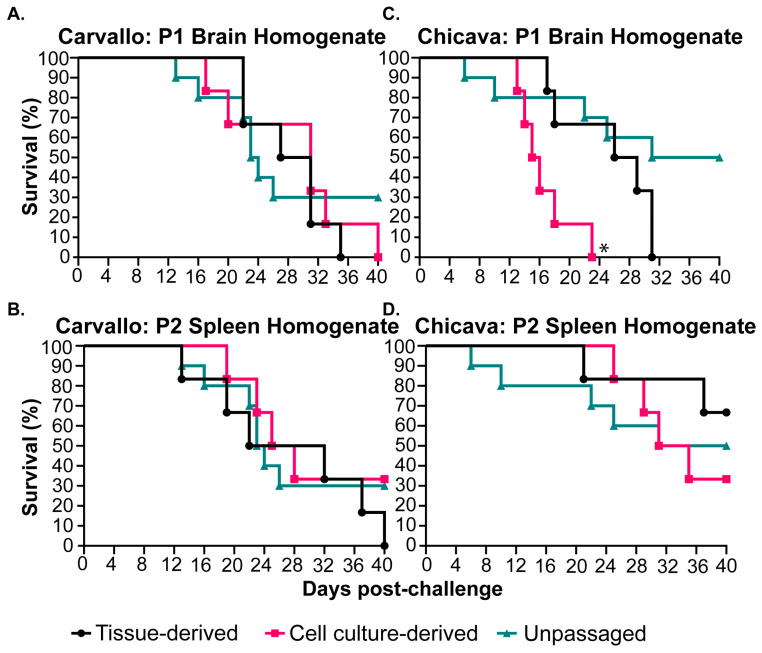
**MACV passaged in brain or spleens of STAT-1^−/−^ mice results in a fully lethal infection.** STAT-1^−/−^ mice (*n* = 10) were infected with 1000 pfu of either (**A**) MACV-Carvallo passaged one time in the brain of STAT-1^−/−^ mice (P1 brain homogenate), (**B**) MACV-Carvallo passaged two times in the spleen of STAT-1^−/−^ mice (P2 spleen homogenate), (**C**) MACV-Chicava passaged one time in the brain of STAT-1^−/−^ mice (P1 brain homogenate), or (**D**) MACV-Chicava passaged two times in the spleen of STAT-1^−/−^ mice (P2 spleen homogenate) via the intraperitoneal route and monitored for survival for 40 days post-challenge. Statistical significance is represented as * *p* ≤ 0.05 relative to the unpassaged isolate.

In total, four stocks were determined to be more lethal than the unpassaged, parental isolates in our preliminary lethality experiments: MACV-Carvallo passaged (i) one time in the brains of STAT-1^−/−^ mice (P1 brain homogenate) MACV-Carvallo passaged (ii) two times in the spleens of STAT-1^−/−^ mice (P2 spleen homogenate); MACV-Chicava passaged (iii) one time in the brains of STAT-1^−/−^ mice (P1 brain homogenate) or MACV-Chicava passaged (iv) two times in the spleens of STAT-1^−/−^ mice (P2 spleen homogenate). To generate viral stocks for use in subsequent in vivo experiments, the tissue-derived virus from the homogenates described above was used to inoculate VeroE6 cells. To confirm lethality of the cell-culture-derived stocks, lethality experiments were conducted with each of the tissue-derived, cell-culture-derived, and wild-type, unpassaged stocks (Figure 4 and Supplementary Figure S3). As expected, challenge with wild-type MACV-Chicava or MACV-Carvallo resulted in partial lethality of 50% and 70%, respectively. Challenge with either the tissue-derived or cell culture-derived stocks of the MACV-Carvallo or MACV-Chicava P1 brain homogenate resulted in complete lethality by day 40 post-challenge (Figure 4A,B), while challenge with the cell culture-derived stock of MACV-Chicava P1 brain homogenate resulted in a faster MTD relative to the tissue-derived and wild-type isolate (16.5 ± 3.62 for cell-culture-derived stock relative to 18.8 ± 10.47 for wild-type isolate). Challenge with the original MACV-Carvallo P2 spleen homogenate resulted in 100% lethality, but the cell culture-derived stock was not fully lethal, and challenge with either the tissue-derived or cell-culture-derived MACV-Chicava P2 spleen homogenate resulted in partially lethal infection comparable to the wild-type MACV-Chicava isolate (Figure 4B,D), but these differences were non-significant. Based on these results, cell-culture-derived stocks of the P1 brain homogenate for both MACV-Carvallo and MACV-Chicava isolates were selected as mouse-adapted isolates for further study [hereafter termed mouse-adapted (ma) MACV-Carvallo and MACV-Chicava].

Machupo virus can be transmitted through inhalation of aerosolized excretions from infected rodents [32,33]. We hypothesized that exposure of mice to MACV through the IN may result in greater lethality of our MACV stocks. Although previous reports indicated that exposure of STAT-1^−/−^ mice to MACV-Chicava through the IN route was less lethal than exposure via the IP route [27], the major differences in disease pathogenesis observed in the model described by Bradfute et al., and the model described here, opens up the possibility that IN challenge may impact disease severity and lethality. To test the impact of IN exposure on lethality, STAT-1^−/−^ mice were challenged with 1000 PFU of either the wild-type MACV-Chicava isolate or maMACV-Chicava by the IP or IN route and monitored for morbidity and mortality for 40 days post-challenge. Challenge with either wild-type or ma MACV-Chicava by the IN route resulted in lower lethality (Supplementary Figure S4A), less weight loss (Supplementary Figure S4B), and more moderate clinicals signs of disease (Supplementary Figure S4C) compared to challenge by the IP route.

### 3.4. Analysis of the Progression of maMACV Disease in STAT-1^−/−^ Mice

#### 3.4.1. Infectious Virus Load

Challenge with maMACV-Chicava resulted in greater lethality and a more rapid clinical course of disease compared to challenge with wild-type MACV (Figure 1 and Figure 2). As such, serial sampling studies were performed to investigate how temporal progression of disease changed following maMACV-Chicava challenge in STAT-1^−/−^ mice. Mice were challenged IP with 1000 pfu of maMACV-Chicava and on days 0, 2, 4, 6, 8, 10, 12, 14, 16, 17, and 18 post-challenge, 3–5 mice were euthanized and their brain, kidney, liver, lung, spleen, testis (where available), and serum were harvested for the analysis of infectious viral load by plaque assay and MACV-specific PCR (Figure 2A). Testes were additionally collected given evidence that MACV can be propagated in rodents through sexual transmission [34]. Given the increased severity of maMACV-Chicava relative to the wild-type isolate, we were unable to collect mice after day 18 post challenge. As expected, a biphasic disease course was observed with mice losing approximately 10–15% of their weight between days 5 and 8, followed by a period where mice recovered weight to baseline, immediately proceeded by rapid weight loss (Supplementary Figure S5A) and severe clinical signs of disease approximately 10–18 days post challenge (Supplementary Figure S5B). Variability in weight loss and clinical score was observed between individual animals; however, variability was less significant compared to that observed with the wild-type isolate (Supplementary Figure S2A). Essentially, all animals had observable clinical signs of disease and exhibited a similar disease progression, as measured by weight loss and clinical scores.

**Figure 5:**
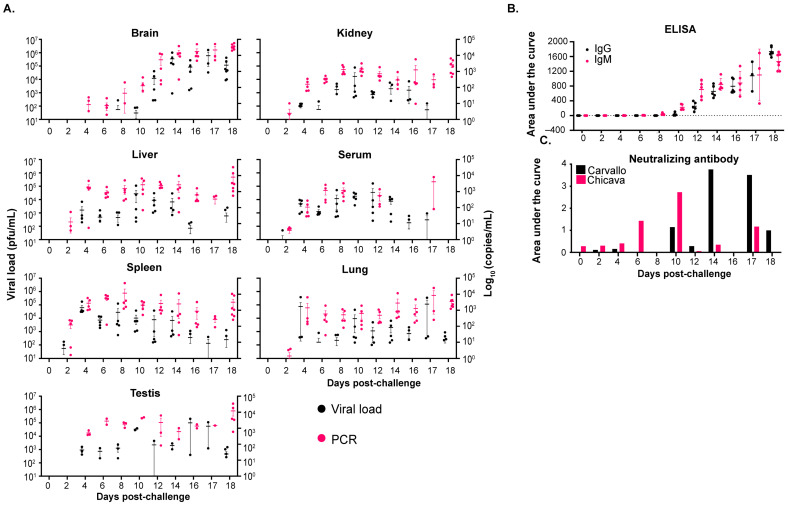
**Disease progression of maMACV-Chicava in STAT-1^−/−^ mice.** STAT-1^−/−^ mice were infected with 1000 pfu of maMACV-Chicava via the intraperitoneal route, and tissue samples were harvested at the indicated time points following MACV infection. (**A**) Samples were evaluated for infectious virus (viral load; black and MACV-specific genome copies by PCR (pink). (**B**) IgG (black)- and IgM (pink)-specific antibodies and (**C**) neutralizing antibodies against MACV-Carvallo (black) and MACV-Chicava (pink) were assessed by ELISA and a microneutralization assay, respectively.

Infectious viral titers were first detected in the spleen and serum on day 2 post challenge, followed by detection in the liver, kidney, and lung on day 4 post challenge (Figure 5A). Titers in these tissues rose slightly overtime, peaking at ~10^5^ pfu/mL 12 days post-challenge, and were detectable throughout the course of the study. The virus was also observed in the testes from day 4 through day 18 post challenge. The virus in the brain was detected by PCR and plaque assay, beginning on days 4 and 8 post challenge, respectively, occurring 6 days prior to the detection of the virus in the brain in animals challenged with wild-type MACV-Chicava (Figure 2), and infectious brain titers continued to increase until the end of the study, approaching ~10^7^ pfu/mL. In general, we observed a similar detection of the virus by PCR in all tissues; however, detection by PCR generally preceded the detection of infectious virus by plaque assay.

#### 3.4.2. Antibody Titers and Neutralizing Response

As before, the IgG- and IgM-specific antibody response was assessed by ELISA (Figure 5B). IgG- and IgM-specific antibodies were initially detected on day 10 post challenge and continued to increase until the end of the study on day 18 post challenge. The development of neutralizing antibodies was assessed by microneutralization assays against MACV-Chicava and MACV-Carvallo (Figure 5C). Significant variability in the development of neutralizing antibody responses was observed over time, likely due to the fact that overall neutralization responses were generally lower than that observed in the wild-type serial sacrifice study (Figure 2C). The first neutralization responses were detected against MACV-Carvallo on day 6 post challenge, but the strongest responses against both isolates were observed between days 14 and 17 post challenge.

#### 3.4.3. Correlation of Viral Load with Weight Change and Clinical Score

Serial sampling studies revealed that infectious virus in the brain can be detected earlier following maMACV challenge compared to challenge with wild-type MACV that persists throughout the course of disease (Figure 2 and Figure 5). Similarly, infection with maMACV-Chicava resulted in mice that lost weight, displayed severe clinical signs of disease and neurological manifestations, and subsequently succumbed to disease earlier than mice challenged with wild-type MACV-Chicava. Given the previous observed relationship between infectious brain titers and measures of morbidity (Table 2 and Table 3), we hypothesized that the faster dissemination of the virus to the brain following maMACV challenge results in enhanced lethality and accelerated disease pathogenesis compared to exposure to wild-type MACV-Chicava. As we did for our previous serial sampling study, correlation coefficients (R^2^) values were calculated for infectious virus from each tissue and individual mouse against the percent of weight loss (Table 4) and clinical score (Table 5) at the time of euthanasia. Like before, significant correlations (R^2^) between brain titer and both weight loss (*p* value 0.0138; R^2^ = 0.1152; Table 4) and clinical score (*p* value 0.0180; R^2^ = 0.1069; Table 5) were observed. A significant correlation between spleen titer and weight loss was also observed; however, this correlation was weaker than the correlation with brain titers. This data provides further evidence of the positive relationship between virus in the brain and the establishment of significant disease and mortality.

#### 3.4.4. Anatomic Pathology

**Figure 6:**
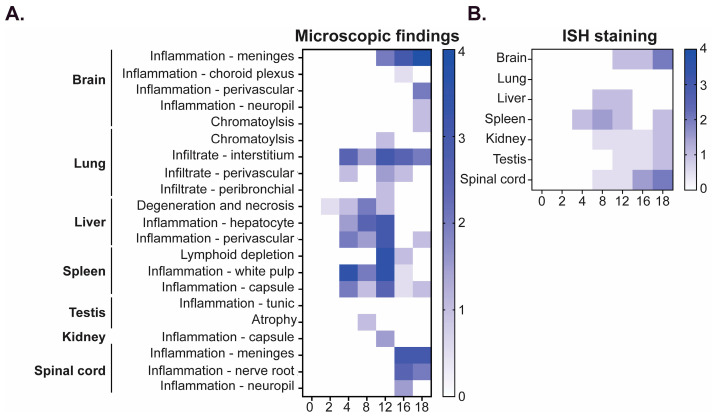
**Pathological changes following maMACV challenge in STAT-1^−/−^ mice.** Heat maps summarizing (**A**) microscopic and (**B**) ISH findings. For (**A**), 0 is no lesion detected; 1 is minimal, with 0–10% of the section affected; 2 is mild, with 11–25% of the section affected; 3 is moderate, with 26–50% of the section affected; and 4 is marked, with >51% of the tissue affected. For (**B**), 0 is no signal present; 1 is <25% of the section having a signal; 2 is 26–50% of the section having a signal; 3 is 51–75% of the section having a signal; and 4 is >75% of the section having a signal.

Histological changes were assessed by H&E (Figure 6A) and ISH staining (Figure 6B). Since the maMACV-Chicava stock was anticipated to be the stock utilized for future efficacy testing, a more comprehensive analysis of pathological changes was undertaken. In general, following challenge with maMACV-Chicava, initial inflammation, histopathological changes, and genomic RNA detection was observed in the liver, spleen, lung, and kidney beginning 2–4 days post challenge, trailed by detection in the brain and spinal cord 8–18 days post challenge (Figure 6A,B).

**Figure 7:**
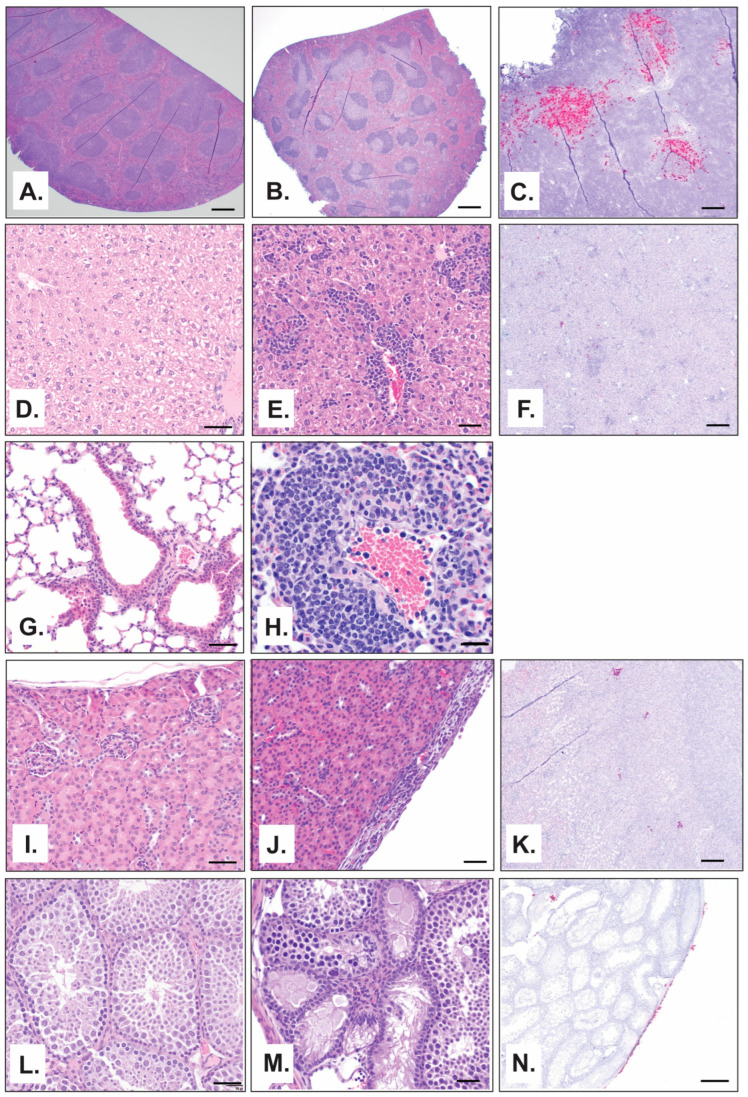
**Pathological changes following maMACV challenge in STAT-1^−/−^ mice in liver, spleen, kidney, lung, and testis.** (**A**,**B**,**D**,**E**,**G**,**H**–**J**,**L**,**M**) Representative pictographs of microscopic findings by H&E. (**C**,**F**,**K**,**N**) Representative pictographs of ISH findings. (**A**–**C**) Spleen, (**D**–**F**) liver, (**G**,**H**) lung, (**I**–**K**) kidney, (**L**–**N**) and testis at (**A**,**D**,**G**,**I**,**L**) day 0, (**B**) day 4, (**E**,**F**,**J**,**M**) day 8, and (**C**,**H**,**K**,**N**) day 12. Scale bar is (**A**,**D**,**G**,**I**,**L**,**E**,**H**,**J**,**M**) 50 µm or (**B**,**C**,**F**,**K**,**N**) 200 µm.

The first significant microscopic findings following challenge was degeneration and necrosis of individual hepatocytes in the liver on day 2 with minimal severity that persisted through day 12 (Figure 7D,E). In most cases, it was accompanied by the infiltration of low-to-moderate numbers of neutrophils and macrophages that expanded around and through vascular walls and inflammation of the capsule by neutrophils and macrophages. Viral antigen was detected by ISH in areas of inflammation, capsule, and individual hepatocytes in the liver on day 8 through 12 and was only evident in a single mouse beyond this period on day 18 (Figure 7F). In the spleen, large areas of lymphoid follicles in the white pulp were depleted of lymphocytes, and infiltration by many neutrophils was observed on day 4 through 12 (Figure 7A,B) with multifocal inflammation of the splenic capsule by neutrophils and macrophages on days 16 to 18. Viral antigen was detected in the spleen by day 4 in the red and white pulp (Figure 6B) and was additionally visible in the capsule on day 18. Although no viral antigen was detected in the lungs by ISH at any time point (Figure 6B), microscopic lesions were observed. The most common microscopic finding in the lung was expansion of the pulmonary interstitium by either neutrophils or a combination of neutrophils, macrophages, and lymphocytes first noted on day 4 (Figure 7G,H). Microscopic lesions in the kidney and testis were minimal. Lesions in the kidney were limited to the infiltration of low numbers of neutrophils and macrophages into the capsule observed in only 14% of mice (*n* = 2) (Figure 7I,J). Despite a general lack of inflammation in the kidney, viral antigen was detected in 36% of mice (36%) beginning on day 8, and was present in the medullary tubules (Figure 7K). In the testis, mild, multifocal atrophy of some seminiferous tubules was observed in one mouse on day 8, and one mouse had moderate neutrophilic and histiocytic inflammation of the tunic covering the testis (Figure 7L,M). On days 12 and 16, viral antigen was detected in a single mouse in the fibrous connective tissue covering of the visceral tunic (Figure 7N) and in the visceral and parietal tunics on day 18.

**Figure 8:**
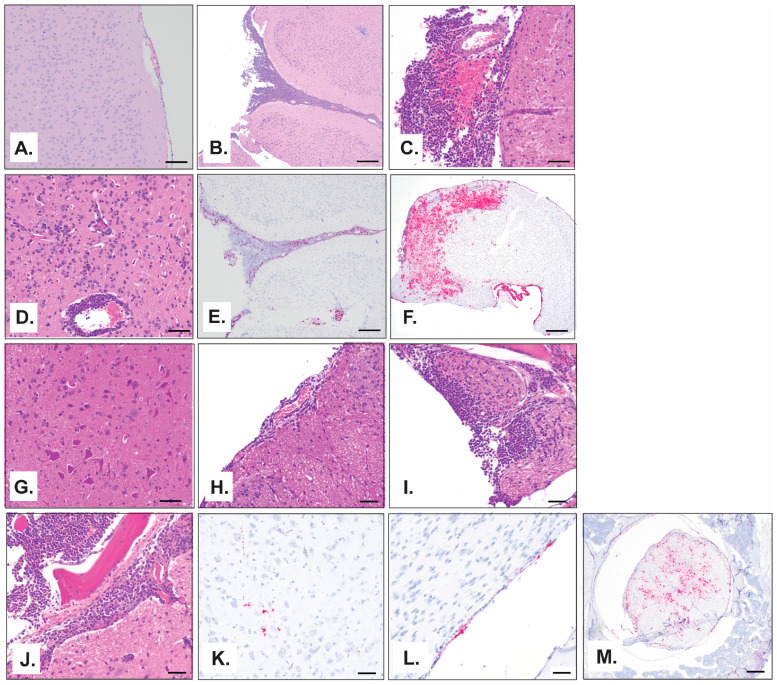
**Pathological changes following maMACV challenge in STAT-1^−/−^ mice in brain and spinal cord.** (**A**–**D**,**G**–**J**) Representative pictographs of microscopic findings by H&E. (**E**,**F**,**K**–**M**) Representative pictographs of ISH findings. (**A**–**F**) Brain and (**G**–**M**) spinal cord at (**A**,**G**) day 0, (**K**,**L**) day 12, (**B**,**E**,**H**,**I**) day 16, and (**C**,**D**,**F**,**J**,**M**) day 18. Scale bar is (**A**,**C**,**D**,**G**–**L**) 50 µm, (**B**,**E**,**M**) 200 µm, or (**F**) 500 µm.

Comparable to the progression of infectious virus described in Figure 5, pathological findings in the brain and spinal cord occurred at later timepoints post-challenge. Lesions were not present in the brain until day 12, which began as multifocal expansion of the leptomeninges with low numbers of neutrophils and macrophages. By day 16, there was a greater number of neutrophils, and a single mouse (of two) had inflammatory cells within the choroid plexus of one of the ventricles (Figure 8A,B). On day 18, inflammatory cells within the leptomeninges (Figure 8C) were greater, and inflammatory cells began to expand to the perivascular space and infiltrate the white and gray matter of the neuropil (Figure 8D). Detection of viral RNA by ISH was noted in the meninges on day 12, which became more widespread on day 16 and included detection in the choroid plexus—the site of cerebrospinal fluid secretion (Figure 8E). Widespread viral antigen was present by day 18 and was present in the meninges, choroid plexus, gray and white matter of the neuropil, and ependymal cells (Figure 8F). Inflammation was observed in the spinal column on day 16 with a similar pathology to that observed in the brain, with many neutrophils and scattered macrophages. The inflammation expanded the meninges (Figure 8G,H), covering the sections of the spinal cord, and extended into the spinal nerve roots (Figure 8I) and portions of the spinal cord itself, continuing through day 18 (Figure 8J). Beginning on day 16, there was also noticeable hyperplasia of granulocytic precursor cells within the bone marrow of the vertebrae in the spinal column sections, reflecting an increased demand for granulocytic leukocytes. Interestingly, although inflammation was not observed in any structure of the spinal column until day 16, viral antigen was detected by ISH in the meninges on day 8 (Figure 6B), and by day 16 a viral signal was visible not only in the meninges but also in the gray matter (Figure 8K) and spinal nerve roots of the spinal cord neuropil (Figure 8L), which was widely dispersed by day 18 (Figure 8M).

#### 3.4.5. Cytokine and Chemokine Analysis

**Figure 9:**
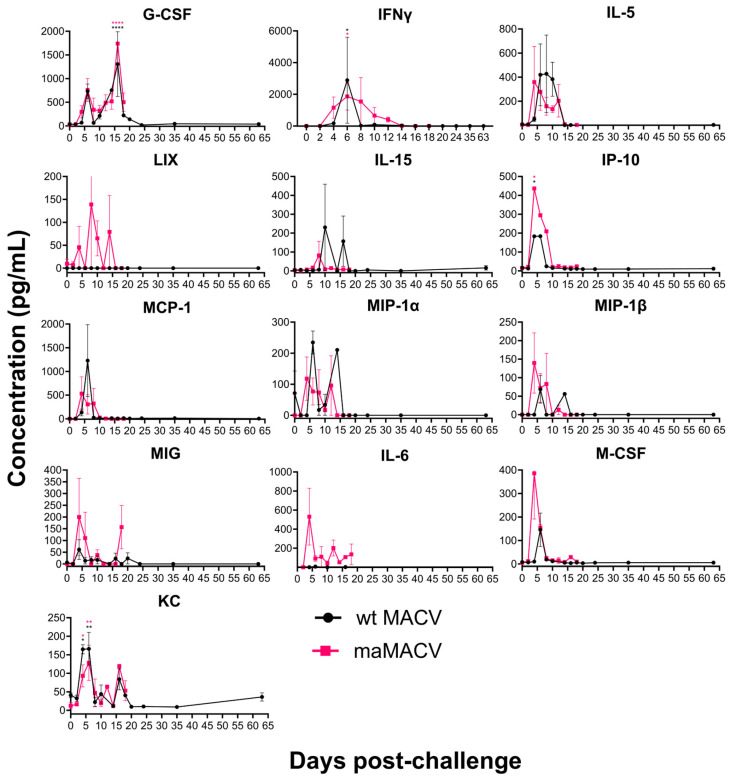
**Cytokine and chemokine analysis.** Levels of 13 different cytokines and chemokines in serum of mice challenged with either wild-type MACV-Chicava (black) or maMACV-Chicava (pink) was evaluated at various times post-infection (*n* = 2–5), and mean fluorescence intensity (MFI) was plotted. Serum samples were collected from serial sacrifice samples described in Figure 2 and Figure 5. Statistical significance is represented by * *p* ≤ 0.05, ** *p* ≤ 0.01, **** *p* ≤ 0.001. Significance is color coded to refer to group and is relative to baseline (day 0).

Cytokine and chemokine levels in the serum from MACV-infected STAT-1^−/−^ mice from both serial sampling studies were analyzed to further discern differences in pathogenesis caused by wild-type and maMACV (Figure 9). The levels of the cytokines/chemokines granulocyte colony-stimulating factor (G-CSF), interferon gamma (IFNγ), interleukin 5 (IL-5), IL-15, interferon-gamma inducible protein 10 (IP-10, CXCL10), monocyte chemoattractant protein 1 (MCP-1, CCL2), macrophage inflammatory protein 1 alpha (MIP-1α, CCL3), MIP-1β (CCL4), monokine induced by interferon-gamma (MIG, CXCL9), macrophage colony-stimulating factor (M-CSF), and keratinocyte chemoattractant (KC, CXCL1) were all elevated relative to day 0 controls between days 2 and 18 post-challenge in mice challenged with either wild-type or maMACV-Chicava (Figure 9). Interestingly, several cytokines/chemokines were increasingly elevated in mice challenged with maMACV-Chicava compared to those challenged with wild-type MACV-Chicava. These cytokines/chemokines included MIG, IFNγ, lipopolysaccharide-induced CXC (LIX, CXCL5), and IL-6 with LIX, IL-6, and IFNγ levels >2 logs higher at various time points in serum from maMACV-Chicava-challenged mice compared to serum from wild-type MACV-Chicava-challenged mice.

#### 3.4.6. Sequencing of MACV and maMACV

Stocks of wild-type and maMACV were sequenced and compared to ascertain genetic differences between the stocks. Surprisingly, no mutations in the large (L) or small (S) segments were detected in the mouse-adapted variants derived from Spleen P2 homogenates. However, mutations in the S segment only were discovered for the mouse-adapted variants derived from the P1 brain homogenates for both isolates. Two non-synonymous mutations in the nucleocapsid protein were observed in the maMACV-Carvallo P1 brain homogenate stock. One mutation resulted in a change from Q to K, and a second mutation resulted in a change from W to a premature stop codon (Table 6). Only one mutation was detected in the maMACV-Chicava P1 brain homogenate stock; however, it was in the intergenic region. All mutations were observed at frequencies between 52.89 and 65.88%.

## 4. Discussion

In this study, STAT-1^−/−^ mice were infected with MACV-Chicava and MACV-Carvallo, resulting in a partially lethal small animal model. Subsequent tissue adaptation in the brains of challenged STAT-1^−/−^ mice resulted in a fully lethal model for both isolates of MACV (Figure 4). Small animal models, including mouse models, are important tools for medical countermeasure development and efficacy testing and can be utilized for rapid down-selection prior to movement to higher-order animal models such as guinea pigs and NHPs. Although a STAT-1^−/−^ mouse model of MACV disease has been described previously, this model resulted in lethality by 8 days post challenge with a lack of neurological disease manifestations. The model described herein differs substantially in that we observed a two-phase disease course and there was no clear and obvious explanation for the disparate results between the two models [27]. Sequencing data confirmed that our stocks of MACV were free of contaminating pathogens and aligned with publicly available sequences of MACV-Chicava and MACV-Carvallo. Differences in sequence identity and cell culture passage history over time between the viral stocks used here and by Bradfute et al. could account for differences in pathogenesis between the two models. Without access to the original stock utilized to compare to the stocks utilized in this report, we are unable to address this possibility through comparative sequence analysis. An additional possibility relates to the source of STAT-1^−/−^ mice. Bradfute et al. utilized STAT-1^−/−^ mice sourced from Taconic Biosciences. Our preliminary studies utilized STAT-1^−/−^ mice from Taconic Biosciences, and, following challenge, we observed a complete absence of lethality, weight loss, and clinical signs of disease (Supplementary Figure S1). However, differences between the STAT-1^−/−^ Taconic strains utilized by Bradfute et al. and in our study could exist. Subsequent studies in STAT-1^−/−^ mice from The Jackson Laboratory provided the lethal model described here. There are slight differences between these two strains. Specifically, STAT-1^−/−^ mice from The Jackson Laboratory were generated utilizing a neo cassette replacing exons 3–5 and part of exon 2 to abolish endogenous gene function and were established in a C57BL/6J background [35], while STAT-1^−/−^ mice from Taconic Biosciences were generated using a neo cassette designed to replace the first three untranslated exons and placed in a 129S6/SvEvTac background [36]. Previous reports have shown that various strains of STAT1-deficieint mice can exhibit differential interferon responses. STAT-1^−/−^ mice lacking the N-terminal domain (NTD-deficient) were shown to be significantly more responsive to IFN compared to mice lacking the DNA-binding domain of STAT-1 (DBD-deficient) [37]. While these lines have been used extensively and interchangeably in many cases, instances where differential susceptibility of these STAT-1^−/−^ strains to viral pathogens have been reported. For example, infection of the NTD-deficient line with herpes simplex virus type 1 (HSV) resulted in transient infection of the liver and spleen, but mice succumbed to encephalitis by day 10 post infection while infection of the DBD-deficient line was rapidly fatal, with significant involvement of the liver and spleen [38]. Differences in the pathogenesis and lethality of other arenavirus species in these two mouse species, including Lassa virus and Lujo virus, have been observed by our group (unpublished), suggesting that the choice of mouse strain, and even vendor, may have dramatic impacts on MACV disease outcomes in STAT-1^−/−^ mice. Regardless, the STAT-1^−/−^ mouse model described here recapitulates the biphasic disease observed in other immunocompromised mouse [26,39], guinea pig [25], and NHP [23,24,30] animal models of MACV disease and recapitulates many aspects of MACV disease observed in humans [5,6,11]. As such, we find it to be a representative model for MACV disease.

Late neurological syndrome is a common complication in humans following MACV infection, which can be lethal or result in temporary or permanent neurological sequelae [5]. We believe that our murine model can become a useful tool to study the neurological syndrome caused by MACV. Although neurological disease for MACV has been described in guinea pigs [25], neurological manifestations are less common than those exhibited in our STAT-1 model. The experimental development of neurological disease for MACV has been described in NHPs, but these are costly and more difficult to handle in the BSL-4 laboratory. Additionally, there is an abundance of immunological and imaging tools available for rodents for further elucidation of neurological disease development that are not available or more difficult to use for NHPs. Continued optimization of this STAT-1^−/−^ model is required to effectively utilize it for antiviral efficacy testing and development.

Interestingly, we observed only two mutations in the nucleoprotein of maMACV-Carvallo compared to the wild-type isolate (Table 6). The arenavirus nucleoprotein (NP) is a multifunctional protein that aids in viral replication and transcription, interacts with the Z protein to recruit ribonucleoprotein complexes into infectious particles, and functions as a type-I IFN antagonist [40,41,42,43,44,45,46,47,48,49,50]. Mutations in NP could modify the inflammatory response following MACV challenge, as well as impact the virus’s replication. While premature stop codons typically lead to non-functional proteins, the consequences can be challenging to decipher considering the complex network of viral protein–host protein interactions that occur during infection and the polyfunctional nature of many viral proteins. More intriguing was the finding that only one mutation was observed between maMACV-Chicava and wild-type MACV-Chicava in the intergenic region (Table 6). Intergenic regions contain regulatory elements like promoters and enhancers that control gene expression levels; therefore, mutations in these regions can contribute to adaptation by altering the regulation of nearby genes and altering the ability of the virus to replicate and cause disease. The adaptation of Marburg virus in rodent species led to a large number of non-coding and/or intergenic region mutations being observed, and further work has shown that mutations in the non-coding region of Ebola virus lead to increased virulence [51,52]. These mutations could also allow the virus to effectively evade the immune system and optimize its replication cycle. Future mechanistic studies to understand the precise molecular functions of these mutations for pathogenesis and adaptation are required. While viral genetic changes play a crucial role in adaptation and pathogenesis, viruses can adapt to different tissue/host environments without detectable sequence alterations, for example by manipulating host epigenetic mechanisms [53,54]. Further investigation is required to understand how serial passaging contributes to greater pathogenesis for these viral isolates.

Infectious virus was detected in the serum, spleen, kidney, and liver at early time points (day 2) following MACV challenge. MACV subsequently spread to the brain on approximately day 12 post challenge, and titers remained high until the time of death (Figure 2). The detection of infectious virus following maMACV was similar; however, the virus was detected more rapidly in the brain, approximately 8 days post challenge. (Figure 5). Histopathological findings mimicked these viral load findings. The most significant histopathological findings were mild-to-moderate liver degeneration and inflammation in the spleen and kidney at early time points (days 2–12) post challenge, followed by severe inflammation and viral genomic RNA detection in the brain and spinal cord at later times post challenge (day 8 through death) (Figure 6). Pathology in the liver, spleen, kidney, lung, and testis was transient, with only minimal pathological findings after day 12 post challenge (Figure 7). However, pathology in the brain and spinal cord increased in severity throughout the course of disease, and viral genome was widely dispersed at terminal study time points (Figure 8). When taken together, viral load data (Figure 2 and Figure 5), correlation analysis (Table 2, Table 3, Table 4 and Table 5), and histopathological findings (Figure 3, Figure 6, Figure 7 and Figure 8) indicate that the dissemination of the virus to the brain and spinal cord triggers the severe morbidity and mortality observed in this STAT-1^−/−^ model. Furthermore, faster dissemination of the virus to the brain and spinal cord appears to account for the enhanced lethality following maMACV challenge (Figure 4).

Almost nothing has been reported on cytokine and chemokine production in response to MACV infection. Here, we show a dysregulated cytokine and chemokine response starting 2 days post challenge and continuing through the course of infection. Increases in the levels of cytokines/chemokines G-CSF, IFNγ, KC, RANTES, MIP-1α, MIP-1β, IL-5, and IL-6 were also observed in the previously reported STAT-1^−/−^ model [27]. The inflammatory response following maMACV challenge was greater than that observed following wild-type MACV challenge, particularly for RANTES, MIG, IFNγ, LIX, and IL-6 (Figure 9). LIX, MIG, and IL-6 are chemokines/cytokines strongly linked to blood–brain barrier (BBB) disruption and recruitment of inflammatory cells to the brain [55,56,57,58]. Increased levels of these cytokines/chemokines have been shown to be associated with viral neurological disease manifestations for dengue and HIV-1 and other neurological conditions such as multiple sclerosis [55,59,60,61]. Furthermore, elevated levels of IL-6 and other cytokines such as IL-8, G-CSF, and TNFα have been observed in the serum of JUNV patients and are correlated with the severity and outcome of disease [62,63,64]. This work provides a reference for the study of cytokine and chemokine production in other MACV animal models.

In conclusion, we report a novel STAT1^−/−^ model of MACV disease that results in fully lethal infection and recapitulates many aspects of disease in NHPs and humans. Further characterization of this model, particularly as it relates to neurological manifestations, is required to understand how it may be utilized for the development of antiviral therapeutics.

## Figures and Tables

**Table 1:** Genbank references.

Genbank Number	Virus	Segment
PV861671	maMACV-Chicava P1 brain	S segment
PV861672	maMACV-Chicava P1 brain	L segment
PV861674	maMACV-Chicava P2 spleen	L segment
PV861675	maMACV-Chicava P2 spleen	S segment
PV861684	maMACV-Carvallo P1 brain	L segment
PV861685	maMACV-Carvallo P1 brain	S segment
PV861680	maMACV-Carvallo P2 spleen	L segment
PV861681	maMACV-Carvallo P2 spleen	S segment

**Table 2:** Correlation of viral load with weight loss following challenge with MACV. Statistical significance is represented as * *p* < 0.05, **** *p* ≤ 0.0001. ns is non-significant.

Tissue	R^2^ vs. Weight Loss	*p*-Value vs. Weight Loss
Brain	0.4169	<0.0001 (****)
Liver	0.00239	0.7498 (ns)
Spleen	0.01828	0.3759 (ns)
Lung	0.1105	0.0257 (*)
Kidney	0.03836	0.1973 (ns)
Serum	0.002975	0.7219 (ns)

**Table 3:** Correlation of viral load with clinical score following challenge with MACV. Statistical significance is represented as * *p* < 0.05, **, *p* < 0.01, **** *p* ≤ 0.0001. ns is non-significant.

Tissue	R2 vs. Clinical Score	*p*-Value vs. Clinical Score
Brain	0.3427	<0.0001 (****)
Liver	0.003746	0.6896 (ns)
Spleen	0.06711	0.0857 (ns)
Lung	0.1962	0.0023 (**)
Kidney	0.125	0.0172 (*)
Serum	0.01172	0.4789 (ns)

**Table 4:** Correlation of viral load with weight loss following challenge with maMACV. Statistical significance is represented as * *p* < 0.05. ns is non-significant.

Tissue	R^2^ vs. Weight Loss	*p*-Value vs. Weight Loss
Brain	0.1152	0.0138 (*)
Liver	0.0064	0.5714 (ns)
Spleen	0.0842	0.0370 (*)
Lung	0.0077	0.5374 (ns)
Kidney	0.0076	0.5376 (ns)
Testis	0.0871	0.1432 (ns)
Serum	0.03588	0.1786 (ns)

**Table 5:** Correlation of viral load with clinical score following challenge with maMACV. Statistical significance is represented as * *p* < 0.05. ns is non-significant.

Tissue	R2 vs. Clinical Score	*p*-Value vs. Clinical Score
Brain	0.1069	0.0180 (*)
Liver	0.0175	0.3504 (ns)
Spleen	0.0584	0.0843 (ns)
Lung	0.00001	0.9788 (ns)
Kidney	0.0002	0.9188 (ns)
Testis	0.1207	0.0821 (ns)
Serum	0.0371	0.1712 (ns)

**Table 6:** Sequencing data.

Virus	Type	Base	SNP	Codon	Feature	Frequency
Carvallo Brain P1	Mutation	C	A	Q (CAA) @247 K (AAA)	Nucleocapsid protein	65.88
	Mutation	G	A	W (TGG) @250 Stop (TGA)	Nucleocapsid protein	61.51
Chicava Brain P1	Mutation	G	A	N/A	Intergenic	52.89

## Data Availability

Data related to this paper may be requested from the authors.

## Supplementary Materials

The following supporting information can be downloaded at https://www.mdpi.com/article/10.3390/v17070996/s1, Figure S1: Wild-type MACV is partially lethal in STAT-1^−/−^ mice from Taconic Biosciences, Figure S2: Weight loss and clinical score in STAT-1^−/−^ mice following MACV-Chicava challenge, Figure S3: Weight loss and clinical score in STAT-1^−/−^ mice following maMACV-Chicava and maMACV-Carvallo challenge, Figure S4: Lethality of MACV-Chicava stocks by IP and IN routes, and Figure S5: Weight loss and clinical scores in STAT-1^−/−^ mice following maMACV-Chicava challenge.
