# Supplementary figures and images for: Characterization of a STAT-1 Knockout Mouse Model for Machupo Virus Infection and Pathogenesis
Source: Viruses. 2025 Jul 16;17(7):996. doi: 10.3390/v17070996 (PMC12299389; doi:10.3390/v17070996)

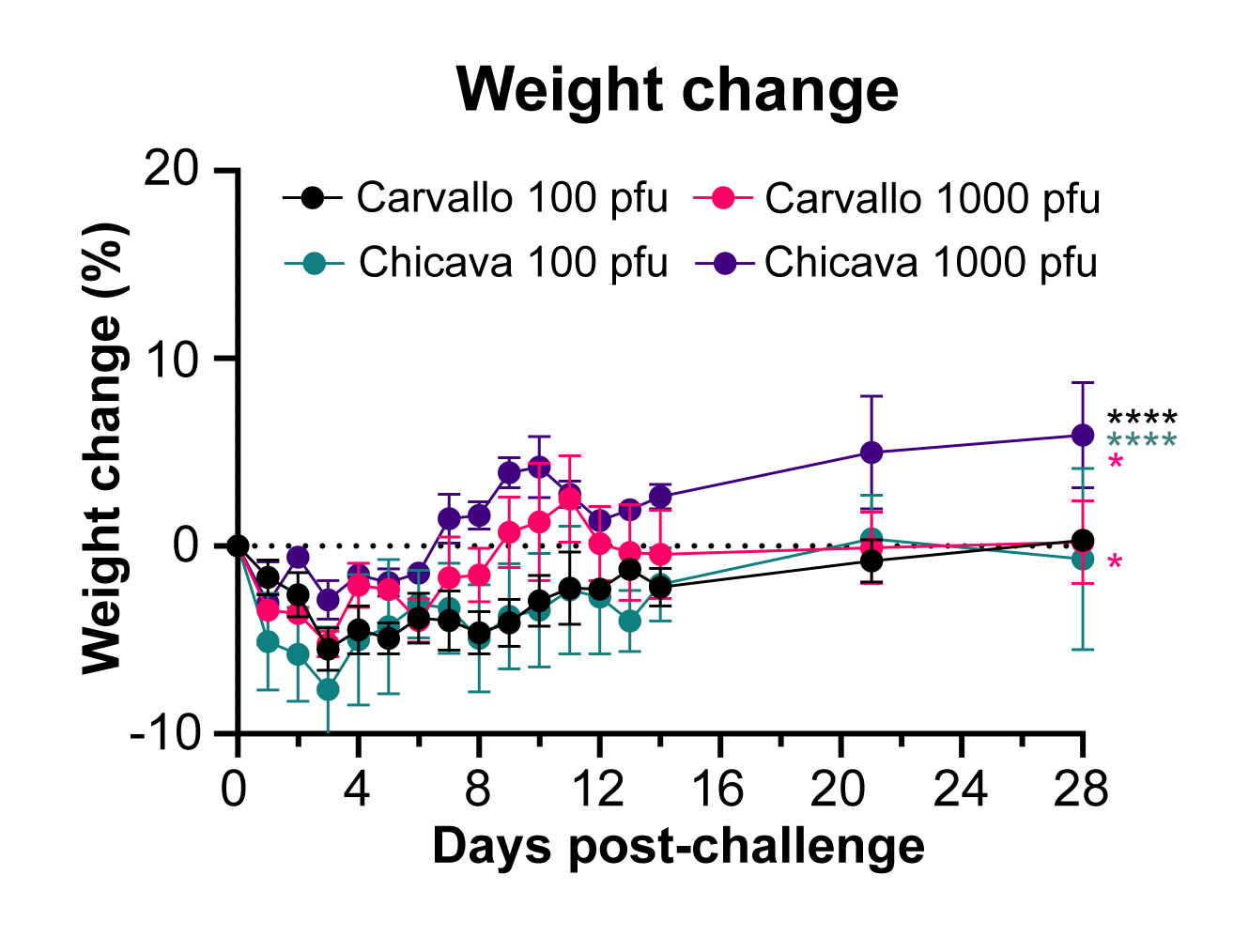

Supplement: Supplementary file 1 [file viruses-17-00996-s001.zip › Supplementary Figure 1.tif]

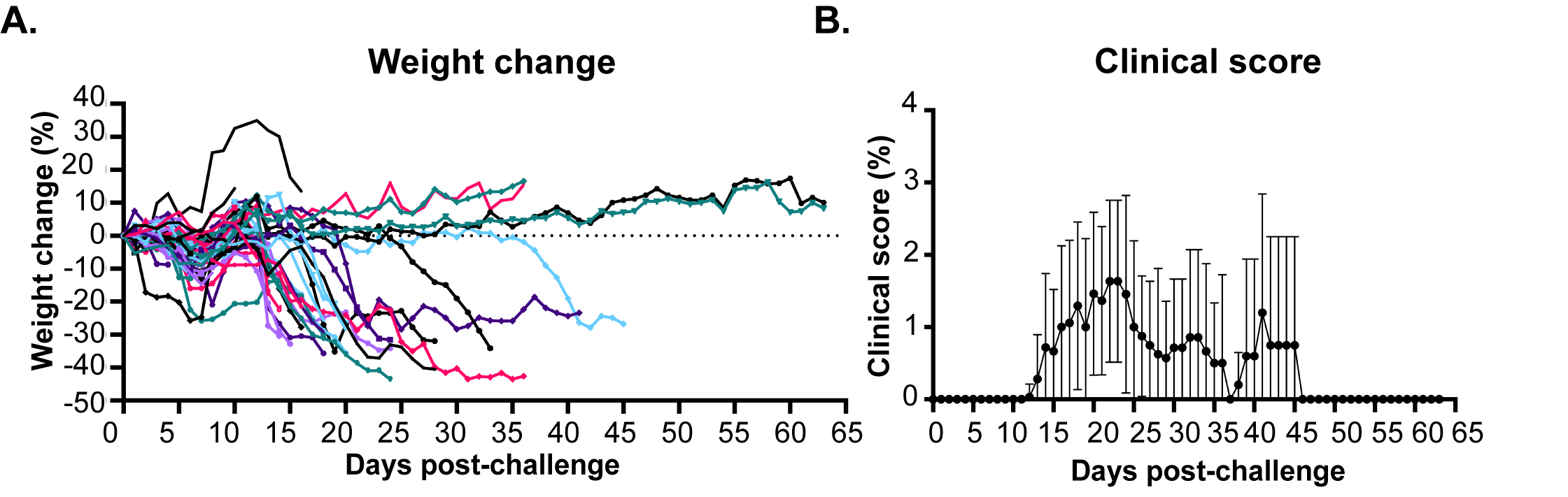

Supplement: Supplementary file 1 [file viruses-17-00996-s001.zip › Supplementary Figure 2.tif]

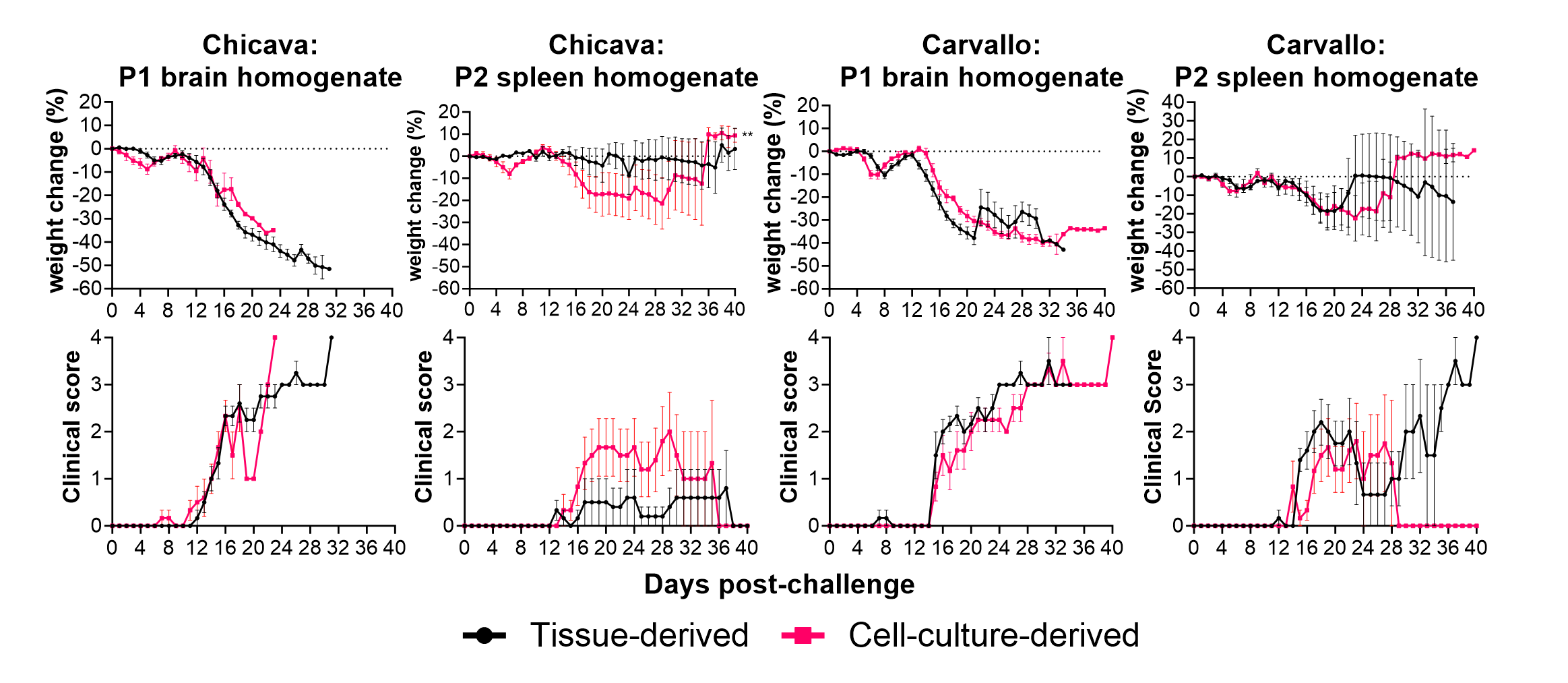

Supplement: Supplementary file 1 [file viruses-17-00996-s001.zip › Supplementary Figure 3.tif]

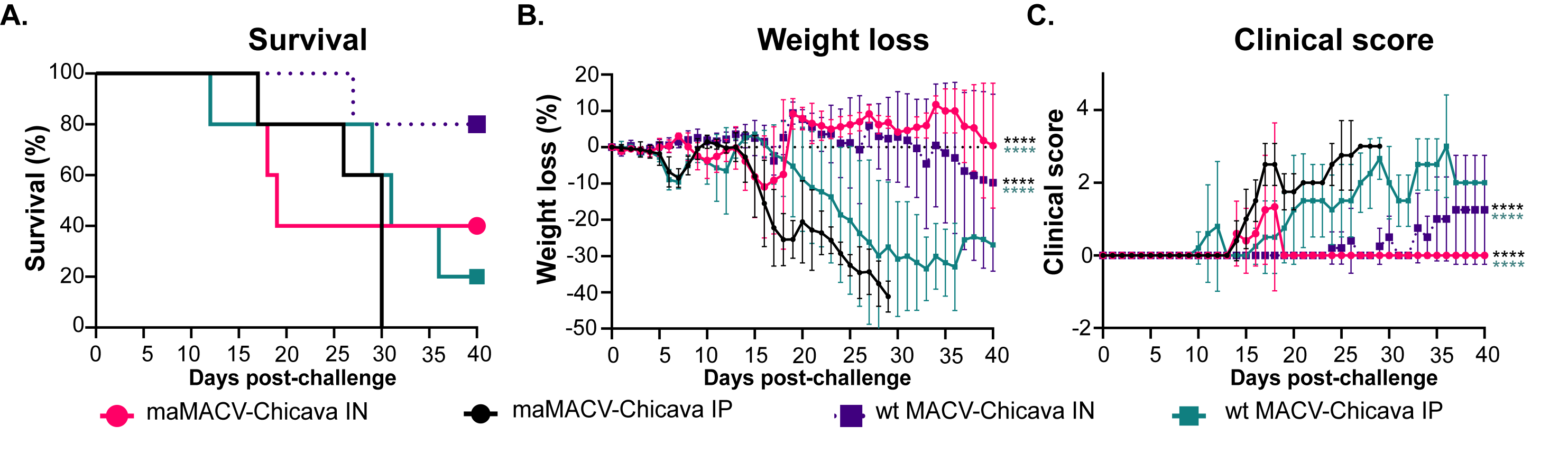

Supplement: Supplementary file 1 [file viruses-17-00996-s001.zip › Supplementary Figure 4.tif]

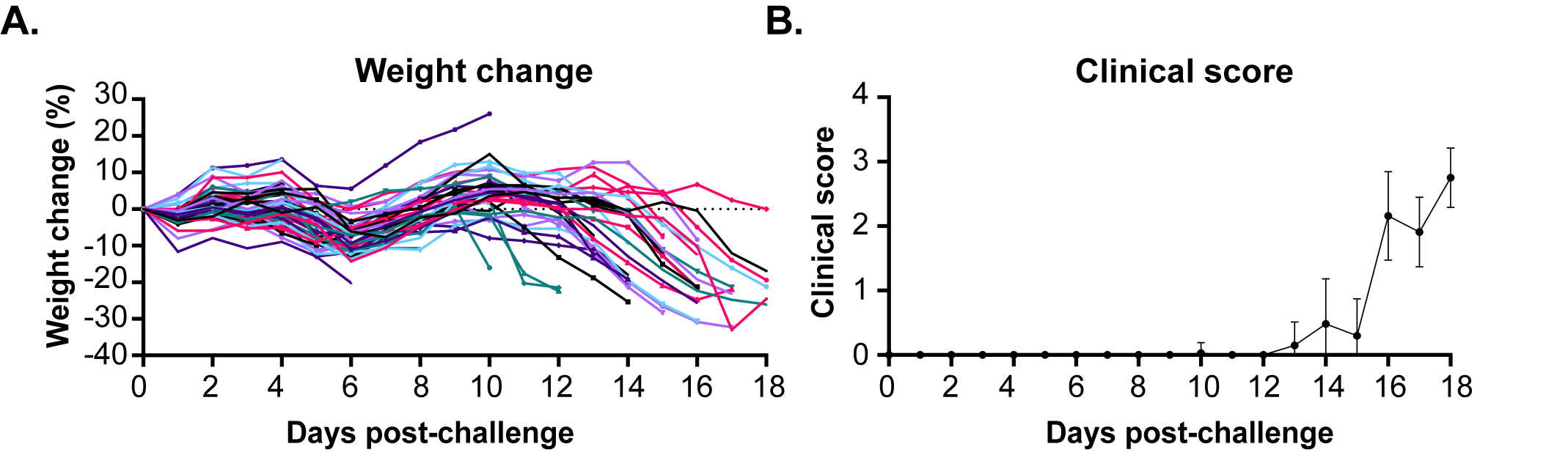

Supplement: Supplementary file 1 [file viruses-17-00996-s001.zip › Supplementary Figure 5.tif]
